# Management of Corneal Clouding in Patients with Mucopolysaccharidosis

**DOI:** 10.3390/jcm10153263

**Published:** 2021-07-24

**Authors:** Orlaith McGrath, Leon Au, Jane Ashworth

**Affiliations:** Manchester Royal Eye Hospital, Central Manchester University Hospitals NHS Foundation Trust, Oxford Road, Manchester M13 9WL, UK; leon.au@mft.nhs.uk (L.A.); jane.ashworth@mft.nhs.uk (J.A.)

**Keywords:** Mucopolysaccharidosis, corneal clouding, penetrating keratoplasty, deep anterior lamellar keratoplasty

## Abstract

Mucopolysaccharidoses (MPS) are a rare group of lysosomal storage disorders characterized by the accumulation of incompletely degraded glycosaminoglycans (GAGs) in multiple organ systems including the eye. Visual loss occurs in MPS predominantly due to corneal clouding and retinopathy, but the sclera, trabecular meshwork and optic nerve may all be affected. Despite the success of therapies such as enzyme replacement therapy (ERT) and hematopoietic stem-cell transplantation (HSCT) in improving many of the systemic manifestations of MPS, their effect on corneal clouding is minimal. The only current definitive treatment for corneal clouding is corneal transplantation, usually in the form of a penetrating keratoplasty or a deep anterior lamellar keratoplasty. This article aims to provide an overview of corneal clouding, its current clinical and surgical management, and significant research progress.

## 1. Introduction

Corneal clouding resulting in photophobia and compromised vision is frequently observed in mucopolysaccharidoses (MPS) subtypes, including MPS I, MPS IV, MPS VI, and MPS VII [1]. While enzyme replacement therapy (ERT) and Hematopoietic stem-cell transplantation (HSCT), improve many of the systemic manifestations of MPS, vision loss remains a significant complication which may adversely affect quality of life. The MPS I registry quantified that over 80% of 302 patients had corneal clouding [2]. Ashworth et al. [1] showed that approximately 80% of 50 patients with MPS type I had visual acuity worse than 6/12 (Snellen) in their better eye and 11% had profound visual impairment (<3/60) in their better eye.

Visual deterioration in MPS occurs due to corneal clouding, retinopathy, glaucoma, and optic neuropathy [1]. (See Table 1 for a summary of the ocular manifestations of MPS). Patients developing retinopathy may complain of a decrease in their peripheral vision and nyctalopia [3]. However, this can be detracted from clinically by visual loss secondary to corneal clouding. Ultimately, the patient develops central visual field loss. Signs on fundus examination may include narrowing of arterioles, foveal external limiting membrane pigmentation/deposition, RPE atrophy, bulls eye maculopathy, and later, bone-spicules [1]. Widespread rod–cone dystrophy can occur [1].

Glycosaminoglycan (GAG) accumulation and consequential corneal thickening can lead to trabecular outflow obstruction and narrowing of the anterior chamber angle. Raised IOP can lead to damage to the optic nerve and glaucoma. In a multicenter case note review study involving four tertiary referral centers, the prevalence of glaucoma ranged from 2.1% to 12.5% in patients with MPS [4]. Peripheral corneal vascularization can develop in patients with MPS, due to blepharitis, exposure, or persistent corneal oedema secondary to raised IOP [5].

Shallow orbits that cause progressive pseudo-exophthalmos, hypertelorism, strabismus, hypermetropia, and astigmatism are common [1]. Non-ocular causes, such as cortical visual impairment, could also result in visual impairment [6]. Graft-versushost disease is a rare complication following HSCT which could lead to conjunctivitis, keratoconjunctivitis sicca, corneal epithelial defects, and pseudo membrane formation [7]. Diagnosing ocular pathologies in patients with MPS can be a challenging task because of physical or mental disabilities which can reduce patient cooperation and tolerance of the exam. If dense, corneal clouding can interfere with accurate ophthalmological examination of the lens, vitreous, and retina [1,5,8,9,10]. Photophobia due to corneal clouding can lead to difficulty tolerating the slit lamp light beam [5].

In patients who have MPS, the frequency of ophthalmic involvement and related visual deterioration requires careful clinical examination, optimal glasses prescription, management of raised IOP and the corneal surface, and, when corneal clouding is the cause of the visual loss, corneal transplantation. 

Corneal clouding treatment remains an unmet need in ophthalmology despite considerable advances in the systemic therapy of MPS. This review article aims to provide an overview of corneal clouding, its current clinical and surgical management, and note-worthy research progress.

## 2. Corneal Clouding in MPS

**Figure 1:**
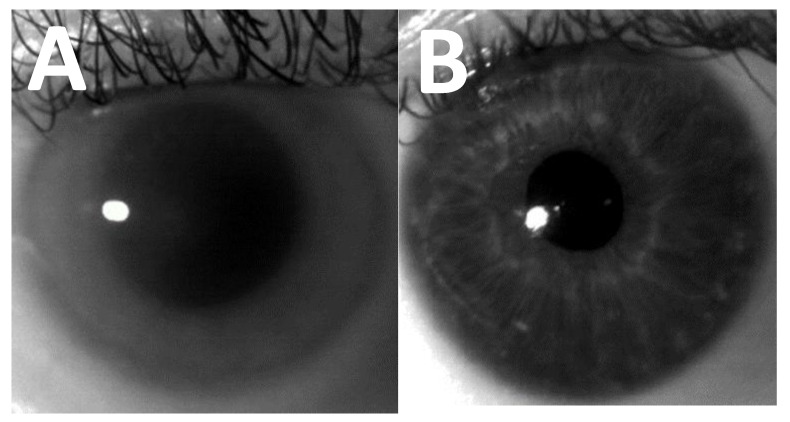
(**A**) Corneal image of a patient with MPS I Hurler demonstrating significant corneal clouding. (**B**) Corneal image of a patient without corneal clouding. Images were taken using IrisGuard AD100, MREH.

While corneal clouding is seen most commonly in MPS I, it is usually most severe in patients with MPS VI (see Figure 1, which demonstrates corneal opacification in MPS I) [1]. It is also seen to some extent in MPS IV, VI, and VII (See Table 1). Corneal clouding is a slowly progressive symptom, therefore visual acuity may be surprisingly good in the early stages of corneal clouding, but subsequently the patient may suffer from photophobia and a slowly progressive loss of visual acuity [6]. It is often present from early childhood [6]. Photochromatic glasses can help to ease symptoms in photophobic patients [11].

Corneal transparency is the outcome of the regular spacing of collagen fibers which have an extraordinarily regular diameter and maintain a consistent interfibrillar space [12,13]. Corneal opacification in MPS is caused by the accumulation of GAGs (see Table 2 which depicts the GAGs that accumulate in the various MPS types), particularly keratan sulphate, in all corneal layers, but particularly within the corneal stroma. The GAG accumulation induces significant light scattering, as the regular arrangement of collagen fibrils that maintain corneal transparency are lost [14,15]. In vivo confocal microscopy typically shows bright intercellular spaces in the corneal layers, extracellular stromal matrix microdeposits, and keratocytes that also have microdeposits and are morphologically modified [15,16]. Corneal clouding affects the entire cornea and it has a characteristic ‘ground glass’ appearance [14]. 

Thickest at the central cornea, the stromal layer is organized predominantly by type I collagens and proteoglycans (a protein core, with GAG side chains composed of either keratin (KS) or dermatan sulphate (DS)) [12]. Keratin proteoglycans manage collagen fibril diameter and dermatan proteoglycans regulate interfibrillar positioning and collagen adhesion [12]. Patients with MPS II are generally spared from corneal clouding [1,6,9,15] (see Table 1.) The deficient enzyme in MPS II (Iduronate-2-sulfatase) results in the build-up of DS containing an additional sulfate group as compared to MPS I and VI. It is thought that the additional sulfate group on the DS in MPS II exhibits a protective effect in the prevention of corneal clouding [17].

GAG deposition in the lysosomes of the keratocytes effects hysteresis, increasing corneal thickness and reducing corneal elasticity, therefore affecting IOP measurements, which rely on normal corneal rigidity for accuracy [4]. Therefore, IOP measurements may be falsely raised in patients with MPS and corneal clouding, potentially leading to difficulty in deciphering whether a raised IOP is secondary to corneal clouding or if it is an indication of potential glaucoma [18,19]. Ashworth et al. found a statistically significant relationship between IOP and the extent of corneal opacification in patients with MPS I and MPS VI [1,6].

## 3. Systemic Therapies and Their Effect on Corneal Clouding

Improvements in quality of life and lifespan due to early systemic treatment have meant that the management of ocular complications and the preservation of vision has increased importance for patients with MPS [1,8]. While systemic treatments significantly improve disease manifestations and prolong life, a considerable burden of disease remains in areas which are not affected by systemic treatments, for example, corneal clouding [17,20].

Early diagnosis of MPS is important, since HSCT is indicated before two years of age and early treatment is associated with better outcomes [21,22,23,24]. Diagnosis is often made after irreversible damage of various tissues has already occurred. Beginning treatment at the asymptomatic stage of the disease has been proven effective at reducing urinary GAGs and organomegaly [23,25]. However, the efficacy of improving functions of the brain and avascular regions, such as the cornea, remains an unmet problem. A key component of more favorable outcomes seems to be starting therapy as early as possible, at diagnosis, before 16 months of age [16,26,27].

There are several sibling studies demonstrating the effectiveness of early treatment on the ocular manifestations of MPS [28]. A 2016 study by Laraway et al. [24,29] studied the outcomes of treatment with ERT in 35 patients with MPS I over a mean study period of 6.5 years. Corneal clouding remained stable in 78% of patients, visual acuity remained stable in 33%, and visual acuity improved in 42%. Younger patients (<10 years at treatment initiation) maintained disease measures closer to norms for age compared with patients aged ≥10 years at treatment initiation [29].

### 3.1. Enzyme Replacement Therapy (ERT) 

ERT provides an impact on most visceral organs, although there is uneven organ biodistribution. ERT has a very limited effect on cornea, bone, central nervous system (CNS), and heart valves, due to the blood–brain barrier. The avascular nature of the cornea means that large proteins are prevented from passing through the blood–cornea or the blood–brain barrier. Despite lowering lysosomal GAG storage amounts to the normal level [30], reports suggest ERT does not seem to prevent progression of corneal or optic disc pathology and, thus, the related worsening of visual function [31]. Photophobia and conjunctival irritation diminish, but corneal clouding and other ocular complications do not usually improve [31,32,33].

There is a paucity of literature about the effect of ERT on corneal clouding specifically. Some studies report an overall stabilizing effect [15,34]; however, other studies describe a worsening of corneal clouding despite treatment [35].

The first ERT clinical trial in 2001 involved 10 patients with MPS I, treated over 52 weeks with weekly intravenous infusions of 0.58 mg/kg laronidase. The extent of corneal clouding did not change in any of the eight patients with corneal clouding. Several patients reported decreased photophobia or conjunctival irritation. Visual acuity improved from 20/1000 to 20/200 (in one eye) in one patient and slightly improved in two others [33].

No changes in corneal opacity were observed by Kakkis and coworkers [33] in 10 patients with MPS I undergoing ERT for one year. Some patients treated with ERT noted an improvement in photosensitivity and corneal clouding, but this was not seen in most [15,29,31,32] Where there are improvements, they are partial and possibly vary among individuals.

In a study by Pitz et al. [31], eight patients with MPS I were followed up for four years while undergoing ERT. One patient had a worsening of VA related to increasing corneal opacity and had subsequent corneal transplantation. Despite repeat immunogenic graft rejections, this patient’s VA improved significantly, from logMAR 0.50 to 0.10, in both eyes. 

A case report by Sarfraz et al. [35] described a patient who was diagnosed with MPS VI at age six. He suffered progressive visual loss and corneal clouding despite being treated with ERT from age 10. His follow up period was over 18 years.

Corneal clouding is difficult to quantify, as it is currently based on the subjective judgement of the clinician. There are also examination difficulties in assessing young children. VA can be more difficult to assess due to comorbid ocular conditions which may be present including glaucoma, optic nerve oedema, optic atrophy, and retinal degeneration [5]. There have recently been studies that demonstrate that corneal densitometry or iris recognition cameras can provide a reliable and objective determination of opacification [19,36,37,38]. These will be useful to determine the effectiveness of treatments in research settings and in clinical practice. ERT’s effect on corneal clouding is limited and variable [20,39].

### 3.2. Hematopoietic Stem-Cell Transplantation (HSCT)

The ocular risks of HSCT include the development of cataracts, epithelial punctate keratopathy, and dry eye syndrome [40,41]. 

Recent studies detected large numbers of myofibroblasts in the cornea of an MPS I patient after HSCT, indicating that corneal clouding may be caused by the transformation of keratocytes into myofibroblasts, which would not be affected by HSCT [20]. 

A 2015 international multi-center study reported on 217 MPS I Hurler patients. Approximately 98% of the patients had corneal clouding before starting HSCT. After treatment, approximately 74% had either stabilized or had shown a decrease in the degree of their corneal clouding [21].

A 2021 study by Guffon et al. [42] analyzed more than 30 years’ worth of data regarding 25 patients with MPS I (Hurler Syndrome) who were treated with HSCT. All 25 patients demonstrated some degree of corneal clouding. In 84%, corneal clouding was diagnosed at a median age of 13 months, before HSCT had begun. Despite HSCT, corneal clouding progressed in all patients and approximately half underwent corneal transplant at a median age of 17.8 years [42]. 

In a retrospective case series, Gullingsrud et al. [43] showed that six patients (30% of a study group of 25 patients with various types of MPS), showed improvements in their corneal clouding, whereas five patients (25%) had worse corneal clouding during follow-up ranging from 7 to 24 months following BMT. No correlation could be made to age at BMT. No eyes with corneal clouding showed complete clouding resolution over the follow up period. One patient required bilateral keratoplasty.

Vellodi and colleagues [44] reported upon two patients with MPS I (Hurler syndrome) who showed complete resolution of corneal clouding after BMT.

Javed et al. carried out a study of nine patients with MPS I (Hurler) or VI (Maroteaux-Lamy) and found that 5/17 (29%) had a significant deterioration in corneal clouding despite ERT or HSCT. One patient whose corneal clouding improved had been treated with ERT from the age of 12 months, before starting HSCT [38]. 

Corneal clouding may progress despite systemic therapy [5]. In a 2018 study by Fahnehjelm et al. [45], which studied eight patients with MPS I Hurler Syndrome, corneal opacities were present in all patients before or shortly after beginning therapy with HSCT. The clouding increased during follow up, despite HSCT, in 5/8 patients. This analysis of ocular disease in a cohort of children with MPS I after HSCT revealed ongoing corneal clouding, as seen in other studies [46]. These studies highlight the need for new MPS treatments that will prevent and reverse corneal clouding.

Sometimes, despite having had systemic therapy from an early age, corneal clouding progresses, impacting on visual acuity and quality of life. A more direct approach is needed to treat ocular pathologies in MPS patients, rather than just ERT and HSCT [46].

## 4. Surgical Treatment for Corneal Clouding

The only current definitive treatment for corneal clouding is corneal transplantation. When the cornea becomes so opacified that vision and quality of life is affected, the patient’s suitability for keratoplasty must be considered. The decision to proceed with a corneal transplant depends on the following factors:

1. The effect of visual impairment on the patient’s daily activities and quality of life, and the wishes of the patient to improve their vision;
2. The exclusion of other ocular factors (retinopathy or optic neuropathy) as a cause of visual impairment;
3. The condition of the ocular surface; dryness or vascularization of the cornea;
4. The general health of the patient and their suitability for anesthesia.

### 4.1. Pre-Operative Planning for Keratoplasty

The decision to undergo a corneal transplant is a joint one between the patient, their family, their ophthalmologist(s), and their metabolic team, based on whether surgical treatment of corneal clouding is in the patient’s best interests. The possibility of graft re-opacification and the risk of only temporary improvements in vision must be thoroughly discussed. Improvements in visual function and quality of life that corneal transplantation can offer to patients with MPS must be weighed against their usually heightened anesthetic risk [9]. Many MPS patients will have concomitant cardiovascular disease, cervical spine instability, short neck, and intubation difficulties. Although local anesthesia may be possible for some types of corneal transplants, very young patients and patients with mental disabilities or behavioral problems may not tolerate surgery without a general anesthetic. This should only be carried out at a specialized center for MPS by an experienced anesthesiologist. Other concomitant eye pathologies must also be taken into careful consideration.

If there is concomitant retinopathy, optic nerve pathology, or glaucoma, visual improvement may be limited, as other factors also affect vision. A corneal transplant might resolve such patients’ corneal opacification, but it will not resolve other ocular issues [47]. Patients should undergo additional tests if possible, such as visual fields, optical coherence tomography (OCT) of the retina and optic nerve, electroretinography (to assess retinal function), and visual evoked potentials (to assess optic nerve function) [5]. If corneal clouding is the principal factor affecting vision, then a corneal transplant can be considered. 

Corneal transplantation demands scrupulous preparation to maximise success. This includes optimisation of the ocular surface condition by use of actions to minimize the harmful effects of ocular surface diseases [48]. Blepharitis, dry eyes, and corneal vascularization may need treatment to reduce the risk of infection and rejection [49]. Preoperative control of glaucoma is also crucial to ensure a successful surgery. Simultaneous cataract surgery along with keratoplasty is something for the surgeon to consider. Glaucoma following keratoplasty is relatively common, it can appear at any period in the evolution of the graft, it can be difficult to diagnose and to monitor and its treatment can hinder the progress of the transplant [50]. Simultaneous cataract surgery and keratoplasty could reduce the risk of anterior chamber angle compromise over time as well as in the immediate post-operative period [51,52]. 

The surgeon must also acknowledge the patient’s postoperative management. Is the patient driven, motivated and suitable for the surgery? The assessment and operation should be carried out in a specialized center for MPS [48]. 

A slit lamp examination (with photographs to help rate the degree of corneal opacification over time) permits the detection of corneal opacities, atypical epithelial corneal changes, or vascularization. It also allows evaluation of the anterior chamber depth to assess for narrow-angle glaucoma. Most studies that evaluate corneal clouding are limited by the subjective results. That is why precise and objective cornea photography methods using the iris camera and Pentacam are useful [36,37]. Corneal topography is of use too [38]. 

Anterior segment optical coherence tomography is useful to measure the corneal thickness and to study the layers of the cornea. It can provide detailed morphological information for the anterior segment in patients with severe corneal clouding [53]. Additionally, in vivo confocal microscopy facilitates thorough examination of corneal cellular changes, assisting in the distinction between stromal and endothelial disease [37].

Tonometry (to measure intra-ocular pressure) and corneal pachymetry (to assess corneal thickness) are important, bearing in mind that intraocular pressure may be falsely raised in patients with MPS [15]. Corneal thickness can be measured using a pachymeter or anterior segment optical coherence tomography (OCT) [54].

Dilated fundus examination could show typical MPS findings such as atrophic optic nerve heads, swollen discs, pathological optic disc cupping, attenuated arterioles, or retinal pigmentary epithelial changes [15]. However, it is important to note that dilation in a phakic patient could result in an attack of acute angle closure. This is because patients with MPS often have occludable angles due to a thickened peripheral cornea and iris [55,56]. A study by Zhang et al. [57] used anterior segment swept-source OCT to analyse anterior chamber angles and found that 5 of 6 patients with MPS I had narrow to closed angles. Even in cases of severe corneal clouding, fundal photographs are recommended as it may be possible to visualise the retina and optic nerve even when this is very difficult via ophthalmoscopy [5,17]. In some cases, examination is hindered by the patient’s photophobia. OCT is important as it could demonstrate cystoid macular oedema, or atrophy of the nerve fibre layer or atrophy of the photoreceptor layer sometimes found in patients with MPS [5].

In patients who have supposed damage to the optic nerve, pattern visual evoked potentials (VEPs) can be useful. Progressive optic nerve swelling and consequent axonal atrophy can lead to trace amplitude reduction [6]. Peak latency increase can result from fiber demyelination caused by the optic nerve compression [15].

Electroretinography (ERG) is recommended in cases of clinical suspicion of retinopathy [1]. ERG recordings in patients with MPS I, II, and III have revealed retinal dysfunctions ranging from none to severe [58]. The ERG abnormalities in patients with MPS show a pattern typical for rod–cone degeneration [1].

There are two main types of corneal transplant, Penetrating Keratoplasty (PK) and Deep Anterior Lamellar Keratoplasty (DALK).

### 4.2. Penetrating Keratoplasty (PK)

PK is the most widely used surgical treatment for corneal opacification. In PK, all five corneal layers are transplanted. Penetrating keratoplasty has been evolving with the use of technology such as femtosecond lasers to generate PKP incisions [59]. An international study of 32 penetrating keratoplasties, performed in patients with MPS and published in 2016, had a graft success rate of 96% [60]. Literature reporting on the PK graft survival rate in patients without MPS indicates a graft survival range of 62% to 86% [61,62]. It is postulated that this higher graft survival rate is because patients with MPS are more receptive to corneal grafts [60]. 

An international 2017 study [60] involving 48 eyes from 32 patients with MPS I, IV, or VI was reported upon. Mean follow-up was 70 months (range: 5–186). PK was performed in 45 eyes and DALK in three eyes. At the last follow-up, a successful visual outcome for PK was found in 63%. Rejection episodes occurred in 23% of grafts; however, a clear graft was recorded at last follow-up in 94% [60]. This study demonstrated that clear corneal grafts can be obtained for patients with corneal clouding due to MPS with improvement in visual acuity in the majority [60]. According to Bothun et al. [47], MPS VI may be associated with a higher graft rejection risk.

Case reports denote that the donor cornea may re-opacify because GAGs can accumulate in donor keratocytes [60,63]. This likely relates to the severity of the MPS and could be due to host keratocyte anterior–posterior spread. There can be gradual replacement of the donor epithelial cells by the host epithelium [9,63]. Käsmann-Kellner et al. [63] suggest that PK may have better outcomes in the MPS types that have dermatan sulphate accumulation (MPS VI, MPS VII) than in the MPS types that have with keratan sulphate accumulation (MPS IV). Dermatan sulphate is present in healed corneal wounds, rejected grafts, and post-viral opacification [63].

Various case reports on MPS patients following PK denote that a clear donor cornea was retained from three months up to five years, without systemic treatment [47,64,65]. A literature review by Bothun et al. described 23 reports that recorded 40 initial and 3 repeat cases of PK in patients with MPS. Thirty-one initial and two repeat corneal grafts were reported as clear, varying from 3 months to 12 years of follow up [47]. A patient with MPS VII had a PK for corneal clouding and this remained clear two years post-transplant [64]. Naumann et al. [65] performed PKs successfully in three children with MPS VI-A (severe type) at the age of 7–11 years. The transplants remained clear during the follow-up over 2.5–5 years and the long-term visual acuity was encouraging. Intriguingly, two of the three patients displayed some clearing of the host cornea adjacent to the transplant. This phenomenon was presumed to have been caused by diffusion of the normal enzyme from the graft to the recipient avascular cornea, to correct the enzyme defect and restore transparency. However, this result has not been reported in any other type of MPS and it could not be duplicated in a study involving a cat model of MPS VI [66].

PK, being an open globe procedure, requires general anesthesia. During corneal transplant surgery, there is often a significant mismatch of corneal thickness between the graft (normal) and the host (abnormally thick). Care should be taken when suturing to allow a smooth anterior corneal surface. This is easier in PK surgery where one can offset the posterior surface but harder to achieve in DALK surgery due to the intact Descemet membrane and the fixed posterior surface [67]. PK is sometimes associated with the loosening of sutures, suture infiltrates, and suture-produced astigmatism [68]. Astigmatism post PK can cause a clear graft to have vision that is not as good as expected [69]. Another important cause of vision poorer than predicted is postopretive glaucoma. Numerous factors can contribute to an increase in the incidence of glaucoma, such as post-operative glaucoma, increased intra-operative manipulation, and severe post-operative inflammation [70]. Rarely, PK can be associated with disastrous problems such as expulsive choroidal hemorrhage [69]. 

### 4.3. Deep Anterior Lamellar Keratoplasty (DALK)

DALK is a partial-thickness cornea transplant that involves the selective transplantation of the epithelium and the stroma, leaving the patient’s native Descemet membrane and endothelium in place. These outer two layers are replaced with a donor epithelium and stroma. In patients with MPS, DALK is often preferred, since corneal clouding is due to GAGs accumulating in the corneal stroma, sometimes sparing Descemet’s membrane and the corneal endothelium. Endothelial involvement occurs only in late stages of MPS, and an intact Descemet’s membrane can act as a barrier to prevent stromal recurrence of MPS in the graft [10]. DALK is contraindicated in patients who have clouding of the endothelium or Descemet’s membrane [60]. DALK may be carried out under local anesthetic in cooperative patients [60]. However, one must take into consideration that conversion to PK may need to happen and good anesthesia and blood pressure control is still very important to avoid complications such as suprachoroidal hemorrhage [71].

A report by the American Academy of Ophthalmology by Reinhart et al. found that there is no advantage to DALK over PK for refractive error outcomes [72]. However, DALK is superior to PK for preservation of endothelial cell density. DALK has an associated lower risk of infection, bleeding, and decreased rates of rejection [69]. Studies comparing the PK procedure to the DALK procedure have demonstrated that DALK can have lower complication rates [73,74]. Because DALK is not a full-thickness procedure, the resultant wound is stronger than that of a PK so the sutures can be removed sooner [72]. Leaving the host endothelium intact considerably reduces the risk of endothelial rejection [75,76]. As DALK is a closed-system operation, there is also decreased risk of immune rejection [76]. Another major DALK advantage is that late corneal failure due to endothelial cell loss, which can be quite common after penetrating keratoplasty, cannot occur after DALK [72]. This is associated with easier postoperative management. Reduced concentration, duration and frequency of topical steroids reduces the risk of steroid-induced glaucoma and cataract [77]. DALK is currently favored over conventional PK in MPS patients due to its similar effectiveness and lower risks [9]. Initially, DALK was performed using manual corneal dissection. Now, DALK can also be performed using a femtosecond laser [67]. Good visual outcomes have been described in patients with conditions such as keratoconus who underwent femtosecond laser-assisted DALK [78].

Phacoemulsification can also be safely performed along with DALK [79]. In patients with MPS, simultaneous keratoplasty and cataract surgery could reduce the risk of post-operative glaucoma [79]. Glaucoma following PK has a relatively high frequency; it can appear early, as well as late, in the evolution of the transplant, it is very hard to diagnose and also to follow-up and the medical or surgical treatment can interfere negatively with the evolution of the corneal graft Over time, there is a risk of anterior chamber angle compromise following DALK. Simultaneous cataract surgery could negate this risk.

Complications are associated with the fundamental difficulty in parting Descemet’s membrane from the stroma [48]. DALK is a more difficult surgery to perform than PK [80]. In patients with MPS, the stroma is more rigid to handle due to GAG deposition which makes the surgical technique of dissection more of a challenge. The poor view due to corneal clouding can pose difficulties for surgeons who prefer a manual dissection technique in DALK surgery. Big bubble technique can be successfully applied but the cloudiness still poses a challenge in gauging the right depth to insert the air needle for big bubble formation [80]. DALK is usually more difficult to perform in younger patients because Descemet’s membrane does not separate as easily [67]. Intra-operative complications include micro perforations and macro perforations of Descemet’s membrane (in which conversion to PK is usually necessary) [48,81]. Post-operative complications include the formation of a double (pseudo-) anterior chamber [67,82]. This is where the donor epithelium separates from the host Descemet’s membrane. It is usually seen in the immediate postoperative period. Risk factors include instances where there was Descemet’s Membrane perforation during surgery [80,83]. To rectify this complication, air is injected surgically into the anterior chamber to tamponade the detached Descemet’s membrane.

Epithelial, stromal, and mixed epithelial and stromal graft rejection occur at a rate of approximately 8 to 10% [84]. Rejection can present any time after three months postoperatively [85]. Graft dehiscence may occur following early suture removal. An interface haze may be apparent in the late post-operative follow-up phase. Haze can occur in cases where stromal fibers have been retained and Descemet’s membrane folds can form as a result [86]. Interface keratitis could occur in the interface, or empty space, that is left. Air or gas in the anterior chamber could lead to pupillary block. 

### 4.4. Postoperative Management

Topical antibiotics and steroid drops should be used during the first post-op month, followed by steroid and lubricant drops for the next six months. After this time period, steroid drop duration is determined by the surgeon’s judgement. Check-ups should be given twice during the first post-operative month, every three months throughout the first post-operative year, and biannually henceforth, until suture removal [15].

Intra- and post-surgical complications are the same as those connected to keratoplasty in any patient. Late complications such as rejection can occur, and it is of paramount importance to assess any case of possible inflammation (red eye and/or visual acuity loss) so that therapy can be administered as early as possible [15]. The eye should be kept completely clean, especially during the first month, and potentially treacherous activities should be prevented to avoid trauma that could cause graft dehiscence. An eye shield can be worn [15].

Re-opacification as early as one-year post graft can occur due to GAG deposition in donor keratocytes [9]. Effectiveness is dependent on the type of GAG deposited and the severity of the disease [63]. Sutures should remain in place for at least one year post-transplantation and following consideration of astigmatism and of the suture condition [5,87]. Suture removal can be done at the slit lamp but may require anesthesia in an uncooperative patient.

## 5. Future Corneal Clouding Treatment Options

### 5.1. Gene Therapy

Gene therapy is currently under development as an MPS treatment option. Gene therapy seems to have the most promise as an effective and permanent solution if it proves to be as safe and effective as seen in animal models. A study published in June 2020 by Miyadera et al. [88] showed that intrastromal gene therapy prevents and reverses advanced corneal clouding in a canine model of MPS I. The eyes with advanced disease demonstrated resolution of corneal clouding as early as one week post-injection, followed by sustained corneal transparency until the experimental endpoint of 25 weeks [88]. This study showed the potential ability of gene therapy to reverse, as well as prevent, corneal clouding. Kamata et al. [89] injected an adenovirus expressing human beta-glucuronidase into the anterior chamber or intrastromal region the cornea of mice who had MPS VII. It successfully treated corneal clouding. Adenovirus-mediated transduction was found throughout the mouse cornea, but it peaked after a few days and then declined after one week.

Vance et al. [90] explored adeno-associated virus gene therapy for alpha-L-iduronidase (IUDA) enzyme delivery by intrastromal injection as a viable remedy for MPS I-related corneal clouding. Seven days after intrastromal injection into human corneas, the enzyme was overproduced in the corneal stroma with extensive dispersal in numerous cell types. There was also a 10-fold escalation in enzyme activity without toxicity.

### 5.2. Substrate Reduction Therapy 

Substrate reduction therapy is expected to affect “difficult-to-treat” tissues such as the cornea and the central nervous system [91]. Small chemical inhibitors are expected to cross the blood–brain barrier as well as the blood–cornea barrier, reaching tissues not accessible to the large recombinant lysosomal enzymes of ERT. Rhodamine B was shown to reduce GAG synthesis in MPS IIIA and VI in vitro studies and in studies of mice with MPS IIIA [91]. Clinical trials are needed to assess the effect on corneal clouding.

In 2006, genistein, a compound that can be purified from soya beans and acts as a tyrosine kinase inhibitor, was identified as showing in vivo reduction of GAG production in MPS I, II, III, VI, and VII fibroblast cells [92]. This drug acts via the epidermal growth factor-dependent pathway to prevent the synthesis of GAGs [93].

Further research needs consistent and repeatable techniques which involve assessing corneal clouding as part of clinical trials to improve current MPS treatment strategies.

## 6. Conclusions

Management of the ocular manifestations of MPS requires a multi-disciplinary approach, with early diagnosis, early initiation of systemic treatment, and careful ocular assessment. Severe corneal clouding may require corneal transplantation. However, novel therapies aim to prevent corneal opacification before the need for surgery arises. Clinical trials need to consistently evaluate the effect of new MPS treatments on corneal clouding to ultimately improve patient outcomes.

## Figures and Tables

**Table 1:** Ocular findings in patients with mucopolysaccharidoses. Data from Ashworth et al. [1].

Disease	Corneal Opacity	Retinopathy	Optic Nerve Abnormalities	Glaucoma
MPS IH Hurler	Very common, mild to severe	Moderate, thickened ELM, parafoveal thinning, parafoveal retinal folds, bulls eye retinopathy	Common, mild to moderate	Uncommon, mild
MPS IH/S Hurler–Scheie	Very common, mild to severe	Moderate, retinal pigment epithelial degeneration	Common, mild to moderate	Uncommon, mild
MPS IS Scheie	Very common, mild to severe	Moderate	Quite common	Uncommon, mild
MPS II Hunter	Rare	Moderate, Pigmented retinopathy	Moderate	Uncommon
MPS III Sanfilippo A-D	Not usually significant	Moderate to severe, with pigmentary retinal degeneration	Rare	Rare
MPS IV Morquio	Some cases, usually mild	Pigmentary retinopathy	Some cases reported	Some cases reported
MPS VI Maroteau x-Lamy	Very common, often severe	Very rare, pigmented retinopathy, parafoveal retinal folds	Common	Unknown frequency
MPS VII Sly	Mild to moderate, can be severe	Unknown frequency	Quite common	Unknown frequency
MPS IX Natowicz	Unknown frequency	Unknown frequency	Unknown frequency	Unknown frequency

**Table 2:** Enzyme defect, glycosaminoglycan deposited and inheritance pattern of the mucopolysaccharidoses. Data from Ashworth et al. [1].

MPS Type	Enzyme Defect	Glycosaminoglycan	Inheritance
MPS IH Hurler	α-L-Iduronidase	Dermatan sulphate, Heparin sulphate	AR
MPS IH/S Hurler–Scheie	α-L-Iduronidase	Dermatan sulphate, Heparin sulphate	AR
MPS IS Scheie	α-L-Iduronidase	Dermatan sulphate, Heparin sulphate	AR
MPS II Hunter	Iduronate-2-sulfatase	Dermatan sulphate, Heparin sulphate	X-linked
MPS IIIA Sanfilippo A	Heparan sulfamidase	Heparin sulphate	AR
MPS IIIB Sanfilippo B	N-Acetyl-α-D-glucosaminidase	Heparin sulphate	AR
MPS IIIC Sanfilippo C	Acetyl-CoA:αglucosaminidase N-acetyltransferase	Heparin sulphate	AR
MPS IIID Sanfilippo D	N-Acetylglucosamine-6-sulfatase	Heparin sulphate	AR
MPS IV Morquio	N-Acetylgalactosamine-6-sulfatase	Keratin sulphate	AR
MPS VI Maroteaux-Lamy	N-acetylgalactosamine-4-sulfatase	Dermatan sulphate,	AR
MPS VII Sly	β-D-Glucuronidase	Dermatan sulphate, Heparin sulphate, Chondroitin sulphate	AR
MPS IX Natowicz	Hyaluronidase	Chondroitin sulphate	AR

AR = autosomal recessive.

## References

[1] Ashworth J.L., Biswas S., Wraith E., Lloyd I.C. (2006). Mucopolysaccharidoses and the Eye. Surv. Ophthalmol..

[2] Pastores G.M., Arn P., Beck M., Clarke J.T., Guffon N., Kaplan P., Muenzer J., Norato D.Y., Shapiro E., Thomas J. (2007). The MPS I registry: Design, methodology, and early findings of a global disease registry for monitoring patients with Mucopolysaccharidosis Type I. Mol. Genet. Metab..

[3] Summers C.G., Ashworth J.L. (2011). Ocular manifestations as key features for diagnosing mucopolysaccharidoses. Rheumatology.

[4] Ashworth J., Flaherty M., Pitz S., Ramlee A. (2015). Assessment and diagnosis of suspected glaucoma in patients with mucopolysaccharidosis. Acta Ophthalmol..

[5] Del Longo A., Piozzi E., Schweizer F. (2018). Ocular features in mucopolysaccharidosis: Diagnosis and treatment. Ital. J. Pediatr..

[6] Ashworth J.L., Kruse F.E., Bachmann B., Tormene A.P., Suppiej A., Parini R., Guffon N. (2010). Ocular manifestations in the mucopolysaccharidoses—A review. Clin. Exp. Ophthalmol..

[7] Nassar A., Tabbara K.F., Aljurf M. (2013). Ocular manifestations of graft-versus-host disease. Saudi, J. Ophthalmol..

[8] Fenzl C., Teramoto K., Moshirfar M. (2015). Ocular manifestations and management recommendations of lysosomal storage disorders I: Mucopolysaccharidoses. Clin. Ophthalmol..

[9] Ferrari S., Ponzin D., Ashworth J.L., Fahnehjelm K.T., Summers C.G., Harmatz P.R., Scarpa M. (2011). Diagnosis and management of ophthalmological features in patients with mucopolysaccharidosis. Br. J. Ophthalmol..

[10] Ganesh A., Bruwer Z., Al-Thihli K. (2013). An update on ocular involvement in mucopolysaccharidoses. Curr. Opin. Ophthalmol..

[11] Fahnehjelm K.T., Malm G., Winiarski J., Törnquist A.-L. (2006). Ocular findings in four children with mucopolysaccharidosis I-Hurler (MPS I-H) treated early with haematopoietic stem cell transplantation. Acta Ophthalmol. Scand..

[12] Michelacci Y.M. (2003). Collagens and proteoglycans of the corneal extracellular matrix. Braz. J. Med. Biol. Res..

[13] Meek K.M., Knupp C. (2015). Corneal structure and transparency. Prog. Retin. Eye Res..

[14] Müller L.J., Pels E., Schurmans L.R., Vrensen G.F. (2004). A new three-dimensional model of the organization of proteoglycans and collagen fibrils in the human corneal stroma. Exp. Eye Res..

[15] Fahnehjelm K.T., Ashworth J.L., Pitz S., Olsson M., Törnquist A.L., Lindahl P., Summers C.G. (2012). Clinical guidelines for diagnosing and managing ocular manifestations in children with mucopolysaccharidosis. Acta Ophthalmol..

[16] Grupcheva C., Craig J.P., McGhee C.N. (2003). In Vivo Microstructural Analysis of the Cornea in Scheie’s Syndrome. Cornea.

[17] Tomatsu S., Pitz S., Hampel U. (2019). Ophthalmological Findings in Mucopolysaccharidoses. J. Clin. Med..

[18] Fahnehjelm K.T., Chen E., Winiarski J. (2012). Corneal hysteresis in mucopolysaccharidosis I and VI. Acta Ophthalmol..

[19] Elflein H.M., Hofherr T., Berisha-Ramadani F., Weyer V., Lampe C., Beck M., Pitz S. (2013). Measuring corneal clouding in patients suffering from mucopolysaccharidosis with the Pentacam densitometry programme. Br. J. Ophthalmol..

[20] Hampe C., Wesley J., Lund T., Orchard P., Polgreen L., Eisengart J., McLoon L., Cureoglu S., Schachern P., McIvor R. (2021). Mucopolysaccharidosis Type I: Current Treatments, Limitations and Prospects for Improvement. Biomolecules.

[21] Aldenhoven M., Wynn R.F., Orchard P.J., O’Meara A., Veys P., Fischer A., Valayannopoulos V., Neven B., Rovelli A., Prasad V.K. (2015). Long-term outcome of Hurler syndrome patients after hematopoietic cell transplantation: An international multicenter study. Blood.

[22] Poe M.D., Chagnon S.L., Escolar M.L. (2014). Early treatment is associated with improved cognition in Hurler syndrome. Ann. Neurol..

[23] Muenzer J. (2014). Early initiation of enzyme replacement therapy for the mucopolysaccharidoses. Mol. Genet. Metab..

[24] Laraway S., Breen C., Mercer J., Jones S., Wraith J.E. (2013). Does early use of enzyme replacement therapy alter the natural history of mucopolysaccharidosis I? Experience in three siblings. Mol. Genet. Metab..

[25] Clarke L.A., Wraith J.E., Beck M., Kolodny E.H., Pastores G.M., Muenzer J., Rapoport D., Berger K., Sidman M., Kakkis E.D. (2009). Long-term Efficacy and Safety of Laronidase in the Treatment of Mucopolysaccharidosis I. Pediatrics.

[26] Giugliani R., Muschol N., Keenan H.A., Dant M., Muenzer J. (2021). Improvement in time to treatment, but not time to diagnosis, in patients with mucopolysaccharidosis type I. Arch. Dis. Child..

[27] De Ru M.H., Boelens J.J., Das A.M., Jones S.A., Van Der Lee J.H., Mahlaoui N., Mengel E., Offringa M., O’Meara A., Parini R. (2011). Enzyme Replacement Therapy and/or Hematopoietic Stem Cell Transplantation at diagnosis in patients with Mucopolysaccharidosis type I: Results of a European consensus procedure. Orphanet. J. Rare Dis..

[28] McGill J.J., Inwood A.C., Coman D., Lipke M.L., De Lore D., Swiedler S.J., Hopwood J.J. (2010). Enzyme replacement therapy for mucopolysaccharidosis VI from 8 weeks of age-a sibling control study. Clin. Genet..

[29] Laraway S., Mercer J., Jameson E., Ashworth J., Hensman P., Jones S. (2016). Outcomes of Long-Term Treatment with Laronidase in Patients with Mucopolysaccharidosis Type I. J. Pediatr..

[30] Gaffke L., Pierzynowska K., Podlacha M., Brokowska J., Węgrzyn G. (2021). Changes in cellular processes occurring in mucopolysaccharidoses as underestimated pathomechanisms of these diseases. Cell Biol. Int..

[31] Pitz S., Ogun O., Bajbouj M., Arash L., Schulze-Frenking G., Beck M. (2007). Ocular Changes in Patients With Mucopolysaccharidosis I Receiving Enzyme Replacement Therapy. Arch. Ophthalmol..

[32] Wraith J.E. (2005). The first 5 years of clinical experience with laronidase enzyme replacement therapy for mucopolysaccharidosis I. Expert Opin. Pharmacother..

[33] Kakkis E.D., Muenzer J., Tiller G.E., Waber L., Belmont J., Passage M., Izykowski B., Phillips J., Doroshow R., Walot I. (2001). Enzyme-Replacement Therapy in Mucopolysaccharidosis I. N. Engl. J. Med..

[34] Pitz S., Ogun O., Arash L., Miebach E., Beck M. (2009). Does enzyme replacement therapy influence the ocular changes in type VI mucopolysaccharidosis?. Graefe’s Arch. Clin. Exp. Ophthalmol..

[35] Sarfraz M.W., Smith M., Jones S., Ashworth J. (2021). Progression of eye disease over 15 years in a patient with mucopolysaccharidosis type VI on enzyme replacement therapy. BMJ Case Rep..

[36] Aslam T., Shakir S., Wong J., Au L., Ashworth J. (2012). Use of iris recognition camera technology for the quantification of corneal opacification in mucopolysaccharidoses. Br. J. Ophthalmol..

[37] Javed A., Aslam T., Ashworth J. (2016). Use of new imaging in detecting and monitoring ocular manifestations of the mucopolysaccharidoses. Acta Ophthalmol..

[38] Javed A., Aslam T., Jones S., Ashworth J. (2017). Objective Quantification of Changes in Corneal Clouding Over Time in Patients With Mucopolysaccharidosis. Investig. Ophthalmol. Vis. Sci..

[39] Summers C.G., Fahnehjelm K.T., Pitz S., Guffon N., Koseoglu S.T., Harmatz P., Scarpa M. (2010). Systemic therapies for mucopolysaccharidosis: Ocular changes following haematopoietic stem cell transplantation or enzyme replacement therapy—A review. Clin. Exp. Ophthalmol..

[40] Fahnehjelm K.T., Törnquist A.-L., Olsson M., Winiarski J. (2007). Visual outcome and cataract development after allogeneic stem-cell transplantation in children. Acta Ophthalmol. Scand..

[41] Fahnehjelm K.T., Winiarski J., Törnquist A.-L. (2008). Dry-eye syndrome after allogeneic stem-cell transplantation in children. Acta Ophthalmol..

[42] Guffon N., Pettazzoni M., Pangaud N., Garin C., Lina-Granade G., Plault C., Mottolese C., Froissart R., Fouilhoux A. (2021). Long term disease burden post-transplantation: Three decades of observations in 25 Hurler patients successfully treated with hematopoietic stem cell transplantation (HSCT). Orphanet. J. Rare Dis..

[43] Gullingsrud E.O. (1998). Ocular abnormalities in the mucopolysaccharidoses after bone marrow transplantation Longer follow-up. Ophthalmology.

[44] Vellodi A., Young E.P., Cooper A., Wraith J.E., Winchester B., Meaney C., Ramaswami U., Will A. (1997). Bone marrow transplantation for mucopolysaccharidosis type I: Experience of two British centres. Arch. Dis. Child..

[45] Fahnehjelm K.T., Olsson M., Chen E., Hengstler J., Naess K., Winiarski J. (2018). Children with mucopolysaccharidosis risk progressive visual dysfunction despite haematopoietic stem cell transplants. Acta Paediatr..

[46] Broek B.T.V.D., van Egmond-Ebbeling M.B., Achterberg J.A., Boelens J.J., Vlessert I.C., Prinsen H.C., van Doorn J., van Hasselt P.M. (2020). Longitudinal Analysis of Ocular Disease in Children with Mucopolysaccharidosis I after Hematopoietic Cell Transplantation. Biol. Blood Marrow Transplant..

[47] Bothun E.D., Decanini A., Summers C.G., Orchard P.J., Tolar J. (2011). Outcome of Penetrating Keratoplasty for Mucopolysaccharidoses. Arch. Ophthalmol..

[48] Tan D.T., Dart J.K., Holland E.J., Kinoshita S. (2012). Corneal transplantation. Lancet.

[49] Pinello L., Busin M., Fontana L., Dua H.S. (2010). Application of (lamellar) keratoplasty and limbal stem cell transplantation for corneal clouding in the mucopolysaccharidoses—A review. Clin. Exp. Ophthalmol..

[50] Ayyala R.S. (2000). Penetrating Keratoplasty and Glaucoma. Surv. Ophthalmol..

[51] Chaurasia S., Price F.W., Gunderson L., Price M. (2014). Descemet’s Membrane Endothelial Keratoplasty. Ophthalmology.

[52] Jones S.M., Fajgenbaum M.A., Hollick E.J. (2015). Endothelial cell loss and complication rates with combined Descemets stripping endothelial keratoplasty and cataract surgery in a UK centre. Eye.

[53] Matoba A., Oie Y., Tanibuchi H., Winegarner A., Nishida K. (2020). Anterior segment optical coherence tomography and in vivo confocal microscopy in cases of mucopolysaccharidosis. Am. J. Ophthalmol. Case Rep..

[54] Ramesh P.V., Jha K.N., Srikanth K. (2017). Comparison of Central Corneal Thickness using Anterior Segment Optical Coherence Tomography Versus Ultrasound Pachymetry. J. Clin. Diagn. Res..

[55] Mullaney P., Awad A.H., Millar L. (1996). Glaucoma in mucopolysaccharidosis 1-H/S. J. Pediatr. Ophthalmol. Strabismus.

[56] Quigley H.A., Maumenee A.E., Stark W.J. (1975). Acute glaucoma in systemic mucopolysaccharidosis I-S. Am. J. Ophthalmol..

[57] Zhang J.R., Wang J.H., Lin H.Z., Lee Y.C. (2020). Anterior Chamber Angles in Different Types of Mucopolysaccharidoses. Am. J. Ophthalmol..

[58] Caruso R.C., Kaiser-Kupfer M.I., Muenzer J., Ludwig I.H., Zasloff M.A., Mercer P.A. (1986). Electroretinographic Findings in the Mucopolysaccharidoses. Ophthalmology.

[59] Price F.W., Price M., Grandin J.C., Kwon R. (2009). Deep anterior lamellar keratoplasty with femtosecond-laser zigzag incisions. J. Cataract. Refract. Surg..

[60] Ohden K.L., Pitz S., Ashworth J., Magalhães A., Marinho D.R., Lindahl P., Fahnehjelm K.T., Summers C.G. (2017). Outcomes of keratoplasty in the mucopolysaccharidoses: An international perspective. Br. J. Ophthalmol..

[61] Williams K.A., Esterman A.J., Bartlett C., Holland H., Hornsby N.B., Coster D.J. (2006). How effective is penetrating corneal transplantation? Factors influencing long-term outcome in multivariate analysis. Transplantation.

[62] Borderie V.M., Boëlle P.Y., Touzeau O., Allouch C., Boutboul S., Laroche L. (2009). Predicted long-term outcome of corneal transplantation. Ophthalmology.

[63] Käsmann-Kellner B., Weindler J., Pfau B., Ruprecht K. (1999). Ocular Changes in Mucopolysaccharidosis IV A (Morquio A Syndrome) and Long-Term Results of Perforating Keratoplasty. Ophthalmology.

[64] Bergwerk K., Falk R.E., Glasgow B.J., Rabinowitz Y.S. (2000). Corneal transplantation in a patient with mucopolysaccharidosis type VII (Sly disease). Ophthalmic Genet..

[65] Naumann G.O., Rummelt V. (1993). Clearing of the para-transplant host cornea after perforating keratoplasty in Maroteaux-Lamy syndrome (type VI-A mucopolysaccharidosis). Klin. Mon. Fur. Augenheilkd..

[66] Aguirre G., Raber I., Yanoff M., Haskins M. (1992). Reciprocal corneal transplantation fails to correct mucopolysaccharidosis VI corneal storage. Investig. Ophthalmol. Vis. Sci..

[67] Nanavaty M.A., Vijjan K.S., Yvon C. (2018). Deep anterior lamellar keratoplasty: A surgeon’s guide. J. Curr. Ophthalmol..

[68] Christo C.G., Van Rooij J., Geerards A.J., Remeijer L., Beekhuis W.H. (2001). Suture-related Complications Following Keratoplasty. Cornea.

[69] Tandon R., Singh R., Gupta N., Vanathi M. (2019). Corneal transplantation in the modern era. Indian J. Med Res..

[70] Al-Mahmood A.M., Al-Swailem S.A., Edward D. (2012). Glaucoma and Corneal Transplant Procedures. J. Ophthalmol..

[71] Chua A., Chua M.J., Kam P. (2018). Recent advances and anaesthetic considerations in corneal transplantation. Anaesth. Intensive Care.

[72] Reinhart W.J., Musch D., Jacobs D., Lee W.B., Kaufman S.C., Shtein R. (2011). Deep Anterior Lamellar Keratoplasty as an Alternative to Penetrating Keratoplasty: A Report by the American Academy of Ophthalmology. Ophthalmology.

[73] Abdelaal A.M., Alqassimi A.H., Malak M., Hijazi H.T., Hadrawi M., Khan M.A. (2021). Indications of Keratoplasty and Outcomes of Deep Anterior Lamellar Keratoplasty Compared to Penetrating Keratoplasty. Cureus.

[74] Janiszewska-Bil D., Czarnota-Nowakowska B., Krysik K., Lyssek-Boroń A., Dobrowolski D., Grabarek B., Wylęgała E. (2021). Comparison of Long-Term Outcomes of the Lamellar and Penetrating Keratoplasty Approaches in Patients with Keratoconus. J. Clin. Med..

[75] Espandar L., Carlson A.N. (2013). Lamellar Keratoplasty: A Literature Review. J. Ophthalmol..

[76] Hos D., Matthaei M., Bock F., Maruyama K., Notara M., Clahsen T., Hou Y., Le V.N.H., Salabarria A.-C., Horstmann J. (2019). Immune reactions after modern lamellar (DALK, DSAEK, DMEK) versus conventional penetrating corneal transplantation. Prog. Retin. Eye Res..

[77] Sharma R.A., Bursztyn L.L., Golesic E., Mather R., Tingey D.P. (2016). Comparison of intraocular pressure post penetrating keratoplasty vs Descemet’s stripping endothelial keratoplasty. Can. J. Ophthalmol..

[78] De Macedo J.P., de Oliveira L.A., Hirai F., De Sousa L.B. (2018). Femtosecond laser-assisted deep anterior lamellar keratoplasty in phototherapeutic keratectomy versus the big-bubble technique in keratoconus. Int. J. Ophthalmol..

[79] Zaki A.A., Elalfy M.S., Said D.G., Dua H.S. (2015). Deep anterior lamellar keratoplasty—Triple procedure: A useful clinical application of the pre-Descemet’s layer (Dua’s layer). Eye.

[80] Ricardo J.R.D.S., Medhi J., Pineda R. (2013). Indications for and Outcomes of Deep Anterior Lamellar Keratoplasty in Mucopolysaccharidoses. J. Pediatr. Ophthalmol. Strabismus.

[81] Jhanji V., Sharma N., Vajpayee R.B. (2010). Intraoperative perforation of Descemet’s membrane during “big bubble” deep anterior lamellar keratoplasty. Int. Ophthalmol..

[82] Karimian F., Feizi S. (2010). Deep Anterior Lamellar Keratoplasty: Indications, Surgical Techniques and Complications. Middle East Afr. J. Ophthalmol..

[83] Basak S.K., Basak S. (2014). Complications and management in Descemet’s stripping endothelial keratoplasty: Analysis of consecutive 430 cases. Indian J. Ophthalmol..

[84] Watson S.L., Tuft S.J., Dart J.K. (2006). Patterns of Rejection after Deep Lamellar Keratoplasty. Ophthalmology.

[85] Perera C., Jhanji V., Lamoureux E., Pollock G., Favilla I., Vajpayee R.B. (2012). Clinical presentation, risk factors and treatment outcomes of first allograft rejection after penetrating keratoplasty in early and late postoperative period. Eye.

[86] Tan D., Ang M., Arundhati A., Khor W.-B. (2015). Development of Selective Lamellar Keratoplasty within an Asian Corneal Transplant Program: The Singapore Corneal Transplant Study (An American Ophthalmological Society Thesis). Trans. Am. Ophthalmol. Soc..

[87] Feizi S., Zare M. (2011). Current Approaches for Management of Postpenetrating Keratoplasty Astigmatism. J. Ophthalmol..

[88] Miyadera K., Conatser L., Llanga T.A., Carlin K., O’Donnell P., Bagel J., Song L., Kurtzberg J., Samulski R.J., Gilger B. (2020). Intrastromal Gene Therapy Prevents and Reverses Advanced Corneal Clouding in a Canine Model of Mucopolysaccharidosis I. Mol. Ther..

[89] Kamata Y., Okuyama T., Kosuga M., O’Hira A., Kanaji A., Sasaki K., Yamada M., Azuma N. (2001). Adenovirus-Mediated Gene Therapy for Corneal Clouding in Mice with Mucopolysaccharidosis Type VII. Mol. Ther..

[90] Vance M., Llanga T., Bennett W., Woodard K., Murlidharan G., Chungfat N., Asokan A., Gilger B., Kurtzberg J., Samulski R.J. (2016). AAV Gene Therapy for MPS1-associated Corneal Blindness. Sci. Rep..

[91] Roberts A.L.K., Thomas B.J., Wilkinson A.S., Fletcher J.M., Byers S. (2006). Inhibition of Glycosaminoglycan Synthesis Using Rhodamine B in a Mouse Model of Mucopolysaccharidosis Type IIIA. Pediatr. Res..

[92] Vadalà M., Castellucci M., Guarrasi G., Terrasi M., La Blasca T., Mule’ G. (2019). Retinal and choroidal vasculature changes associated with chronic kidney disease. Graefe’s Arch. Clin. Exp. Ophthalmol..

[93] Jakóbkiewicz-Banecka J., Piotrowska E., Narajczyk M., Barańska S., Węgrzyn G. (2009). Genistein-mediated inhibition of glycosaminoglycan synthesis, which corrects storage in cells of patients suffering from mucopolysaccharidoses, acts by influencing an epidermal growth factor-dependent pathway. J. Biomed. Sci..

